# Mental Health and Challenges of Family Caregivers: A Public Health Perspective in India

**DOI:** 10.1002/alz70858_099703

**Published:** 2025-12-24

**Authors:** Istikhar Ali, Aparajit Ballav Dey, Shailendra Singh Bhadouria

**Affiliations:** ^1^ Jawaharlal Nehru University, South Delhi, Delhi, India; ^2^ Venu Geriatric Institute, South Delhi, Delhi, India; ^3^ All India Institute of Medical Sciences, New Delhi, Delhi, India

## Abstract

**Background:**

India's rapidly aging population necessitates long‐term care for elderly individuals, a responsibility predominantly borne by family caregivers. While caregiving is culturally valued, it imposes substantial mental, physical, and financial burdens, which are further exacerbated by inadequate systemic support.

**Method:**

This study explores the challenges faced by family caregivers in India through a mixed‐methods approach, incorporating data from the interRAI Family Carer Needs Assessment (FCNA) tool and Focus Group Discussions (FGDs).

**Result:**

The findings reveal high levels of psychological distress, with 60% of caregivers experiencing anxiety and 50% reporting sleep disturbances. Complementing these quantitative insights, the study provides a qualitative exploration of caregivers' daily lives, highlighting the multidimensional nature of caregiving. Caregivers face emotional exhaustion, social isolation, and role strain, compounded by systemic barriers such as the lack of formal support structures, financial strain, and limited respite care services.

**Conclusion:**

This study underscores the urgent need for comprehensive policies, systemic reforms, and increased societal engagement to alleviate caregiver burdens and ensure sustainable elder care. These findings lay the groundwork for developing scalable, culturally sensitive interventions aimed at improving caregiver well‐being and enhancing patient outcomes.

